# Supplementary material for: Concentrations, Possible Sources and Health Risk of Heavy Metals in Multi-Media Environment of the Songhua River, China
Source: Int J Environ Res Public Health. 2020 Mar 9;17(5):1766. doi: 10.3390/ijerph17051766 (PMC7084879; doi:10.3390/ijerph17051766)
Supplement: Supplementary file 1 [file ijerph-17-01766-s001.pdf]

Supplementary Information

Table S1. Distribution of sampling sites of the Songhua River.

Name	longitude（E）	latitude (N)	Site
S1	124°48'28"	45°10'2"	Songyuan（Estuary）
S2	125°5'57"	45°44'57"	Zhanyuan（Estuary）
S3	125°41'31"	45°27'13"	Lalinhe（Estuary）
S4	126°31'11"	45°46'10"	Harbin（City）
S5	126°42'2"	45°48'55"	Harbin（City）
S6	127°32'37"	46°2'29"	Bayan（Cultivated field）
S7	128°2'26"	45°56'22"	Mulan（Town）
S8	128°45'60"	45°58'22"	Tonghe（Town）
S9	129°32'12"	46°19'48"	Yilan（Town）
S10	129°57'25"	46°44'54"	Tangyuan（Cultivated field）
S11	130°15'57"	46°50'3"	Jiamusi（City）
S12	130°24'45"	46°50'32"	Jiamusi（City）
S13	131°57'23"	47°14'7"	Fujin（Cultivated field）
S14	132°30'54"	47°41'27"	Tongjiang（Estuary）

Table S2. Classifications of heavy metal pollution degree in drinking water.

HPI	Degree of pollution [1]
$HPI < 15$	low
$15 \leq HPI < 30$	medium
$30 \leq HPI < 100$	high

Table S3. Classification of heavy metal pollution degree.

P_i	P_N	Degree of pollution [2,3]
$P_i \leq 1$	$P_N \leq 1$	nonpollution
$1 < P_i \leq 2$	$1 < P_N \leq 2.5$	Light pollution
$2 < P_i \leq 3$	$2.5 < P_N \leq 7$	Moderate pollution
$P_i > 3$	$P_N > 7$	Severe pollution

Table S4. Worldwide average concentration of trace elements in sediment from major river of the world (mg kg^{-1}).

River	Cu	Cr	Zn	Pb	Ni	Cd	References
Songhuajiang (China)	14.6	64.0	175.8	16.8	22.7	0.28	This study
Yellow river (China)	40.7	62.4	68.4	15.2	23.6	0.085	[4]
Ebro River (Portugal)	21.8	34.3	83.5	15.8	13.7	0.3	[5]
Seine River (France)	14	52	76	26	27	0.3	[6]
Yangtze river (China)	30.7	78.9	94.3	27.3	31.8	0.26	[7]
Xiangjiang (China)	35.16	38.17	346.17	111.83	-	15.28	[8]
Brisbane River (Australia)	20-110	82-332	142-257	25-126	20-34	0.6-0.9	[9]
Shur River (Iran)	9174	-	522	162	-	6.85	[10]
Urμguay River (Argentina)	55	19	85	13	16	-	[11]
Jiaozhou Bay (China)	23.6	69.3	64.6	20.2	-	0.159	[12]
Nile River (Egypt)	-	173	74	-	48	0.3	[13]

Table S5. The single contamination factor (*Pi*) of sediment and riparian soil in Songhua River.

Sampling site	Cu		Cr		Zn		Pb		Ni		Cd	
	Sediment	Soil	Sediment	Soil	Sediment	Soil	Sediment	Soil	Sediment	Soil	Sediment	Soil
S1	1.19	0.83	1.52	1.17	3.24	2.29	0.97	0.78	0.99	1.02	5.24	3.22
S2	0.74	1.05	1.45	1.38	2.22	1.82	0.89	0.74	0.83	1.34	2.91	1.88
S3	0.49	0.54	1.08	1.01	1.42	1.64	0.65	0.72	0.97	0.72	1.91	3.08
S4	0.92	0.59	1.50	0.82	1.83	1.15	0.71	0.38	1.47	0.62	2.87	3.27
S5	0.62	0.89	0.84	0.29	1.69	1.96	0.64	0.65	0.72	0.73	1.48	3.85
S6	0.59	1.54	0.93	1.25	2.04	2.41	0.33	1.17	0.94	1.06	2.69	6.82
S7	0.71	1.26	0.82	1.51	2.89	1.80	0.33	0.82	0.99	1.31	3.56	2.27
S8	0.61	0.96	0.57	1.27	1.70	1.48	0.69	0.88	0.89	1.37	1.36	5.51
S9	1.14	0.97	0.96	1.62	2.60	1.95	0.73	0.89	1.55	1.14	6.79	2.62
S10	0.49	0.74	0.99	1.37	4.61	3.13	0.72	0.80	0.88	1.04	3.69	10.15
S11	0.40	0.57	0.92	1.13	1.77	1.64	0.77	0.70	0.59	1.33	3.02	1.50
S12	0.73	1.00	1.18	1.54	4.10	1.88	0.76	0.94	1.15	0.92	5.04	2.76
S13	0.62	0.97	1.09	1.81	2.29	2.12	0.79	0.65	0.84	0.78	2.02	2.85
S14	0.96	0.88	1.42	1.56	2.38	3.61	0.77	0.76	1.14	1.23	3.20	0.43
Mean	0.73	0.91	1.09	1.27	2.49	2.06	0.70	0.78	1.00	1.04	3.27	3.59

Table S6. Pearson correlation matrix for heavy metals in surface sediments of the Songhua River.

	Cu	Cr	Zn	Pb	Ni	Cd
Cu	1					
Cr	0.689**	1				
Zn	0.225	0.402	1			
Pb	0.266	0.322	0.217	1		
Ni	0.729**	0.953**	0.502	0.361	1	
Cd	-0.022	-0.017	0.035	0.024	0.063	1

** Correlation is significant at $p < 0.01$ level (two-tailed);

References

1. Edet, A.E.; Offiong, O.E.; Evaluation of water quality pollution indices for heavy metal contamination monitoring A study case from Akpabuyo-Odukpani area, Lower Cross River Basin (southeastern Nigeria). *GeoJournal*. **2002**, *57*, 295-304.
2. Nemerow, N.L.C. Scientific Stream Pollution Analysis. Scripta Book Company： Washington, DC, USA, 1974.
3. Shaheen, A.; Iqbal, J.; Hussain, S. Adaptive geospatial modeling of soil contamination by selected heavy metals in the industrial area of Sheikhupura, Pakistan. *Int. J. Environ. Sci. Te.* **2019**, *16,* 4447-4464.
4. Yan, N.; Liu, W.; Xie, H.; Gao, L.; Han, Y.; Wang, M.; Li, H. Distribution and assessment of heavy metals in the surface sediment of Yellow River, China. *J. Environ. Sci-China* **2016**, *39*, 45-51.
5. Roig, N.; Sierra, J.; Moreno-Garrido, I.; Nieto, E.; Gallego, E.P.; Schuhmacher, M.; Blasco, J. Metal bioavailability in freshwater sediment samples and their influence on ecological status of river basins. *Sci. Total. Environ.* **2016**, *540*, 287-296.
6. LeCloatec, M.F.; Bonete, P.H.; Lestel, L.I.; Ayrault, S. Sedimentary record of metal contamination in the Seine River during the last century. *Phys. Chem. Earth.* **2011**, *36*, 515-529.
7. Zhang, W.; Feng, H.; Chang, J.; Qu, J.; Xie, H.; Yu, L. Heavy metal contamination in surface sediments of Yangtze River intertidal zone: An assessment from different indexes. *Environ. Pollut.* **2009**, *157*, 1533-1543.
8. Liu, J., Xu, Y.; Cheng, Y.; Zhao, Y.; Pan, Y.; Fu, G.; Dai, Y. Occerrence and risk assessment of heavy metals in sediments of the Xiangjiang River. China. *Environ. Pollut.* **2017**, *24*, 2711-2723.
9. Duodu, G.O.; Goonetilleke, A.; Ayoko, G.A. Comparison of pollution indices for the assessment of heavy metal in Brisbane River sediment. *Environ. Pollut.* **2016**, *219*, 1077-1091.
10. Karbassi, A.R.; Monavari, S.M.; Bidhendi, G.R.N.; Nouri, J.; Nematpour, K. Metal pollution assessment of sediment and water in the Shur River. *Environ. Monit. Assess.* **2008**, *147,* 107-116.
11. Tatone, L.M.; Bilos, C.; Skorupka, C.N.; Colombo, J.C. Comparative approach for trace metal risk evaluation in settling particles from the Uruguay River, Argentina: enrichment factors, sediment quality guidelines and metal speciation. *Environ. Earth Sci.* **2016**, *75,* 575.
12. Xu, F.; Liu, Z.; Cao, Y.; Qiu, L.; Feng, J.; Xu, F.; Tian, X. Assessment of heavy metal contamination in urban river sediments in the Jiaozhou Bay catchment, Qingdao, China. *Catena* **2017**, *150*, 9-16.
13. Badawy, W.M.; Ghanim, E.H.; Duliu, O.G.; El Samman, H.; Frontasyeva, M.V. Major and trace element distribution in soil and sediments from the Egyptian central Nile Valley. *J. Afr. Earth Sci.* **2017**, *131*, 53-61.
